# Metabolic Alterations in Sputum and Exhaled Breath Condensate of Early Stage Non-Small Cell Lung Cancer Patients After Surgical Resection: A Pilot Study

**DOI:** 10.3389/fonc.2022.874964

**Published:** 2022-06-03

**Authors:** Naseer Ahmed, Biniam Kidane, Le Wang, Zoann Nugent, Nataliya Moldovan, April McElrea, Shiva Shariati-Ievari, Gefei Qing, Lawrence Tan, Gordon Buduhan, Sadeesh K. Srinathan, Renelle Meyers, Michel Aliani

**Affiliations:** ^1^CancerCare Manitoba Research Institute, Winnipeg, MB, Canada; ^2^Department of Radiology, Section of Radiation Oncology, Rady Faculty of Health Sciences, University of Manitoba, Winnipeg, MB, Canada; ^3^Department of Physiology and Pathophysiology, Rady Faculty of Health Sciences, University of Manitoba, Winnipeg, MB, Canada; ^4^Department of Surgery, Rady Faculty of Health Sciences, University of Manitoba, Winnipeg, MB, Canada; ^5^Department of Epidemiology and Cancer Registry, CancerCare Manitoba, Winnipeg, MB, Canada; ^6^St. Boniface Hospital Albrechtsen Research Centre, Winnipeg, MB, Canada; ^7^Department of Pathology, Rady Faculty of Health Sciences, University of Manitoba, Winnipeg, MB, Canada; ^8^Department of Surgery, University of British Columbia, Vancouver, BC, Canada; ^9^BC Cancer Research Institute, University of British Columbia, Vancouver, BC, Canada; ^10^Department of Food and Human Nutritional Sciences, University of Manitoba, Winnipeg, MB, Canada

**Keywords:** lung, cancer, metabolism, surgical, resection

## Abstract

Every year, close to two million people world-wide are diagnosed with and die of lung cancer. Most patients present with advanced-stage cancer with limited curative options and poor prognosis. Diagnosis of lung cancer at an early stage provides the best chance for a cure. Low- dose CT screening of the chest in the high-risk population is the current standard of care for early detection of lung cancer. However, CT screening is invasive due to radiation exposure and carries the risk of unnecessary biopsies in non-cancerous tumors. In this pilot study, we present metabolic alterations observed in sputum and breath condensate of the *same* population of early- stage non-small cell lung cancer (NSCLC) patients cancer before and after surgical resection (SR), which could serve as noninvasive diagnostic tool. Exhaled breath condensate (EBC) (n=35) and sputum (n=15) were collected from early-stage non-small cell lung cancer (NSCLC) patients before and after SR. Median number of days for EBC and sputum collection before and after SR were 7 and 42; and 7 and 36 respectively Nuclear magnetic resonance (NMR) and liquid chromatography quadrupole time-of-flight mass spectrometry (LC-QTOF-MS) were used to analyze the metabolic profile of the collected samples. A total of 26 metabolites with significant alteration post SR were identified, of which 14 (54%) were lipids and 12 constituted nine different chemical metabolite classes. Eighteen metabolites (69%) were significantly upregulated and 8 (31%) were downregulated. Median fold change for all the up- and downregulated metabolites (LC-QTOF-MS) were 10 and 8, respectively. Median fold change (MFC) in concentration of all the up- and downregulated metabolites (NMR) were 0.04 and 0.27, respectively. Furthermore, glucose (median fold change, 0.01, p=0.037), adenosine monophosphate (13 log fold, p=0.0037) and N1, N12- diacetylspermine (8 log fold p=0.011) sputum levels were significantly increased post-SR. These identified sputa and EBC indices of altered metabolism could serve as basis for further exploration of biomarkers for early detection of lung cancer, treatment response, and targets for drug discovery. Validation of these promising results by larger clinical studies is warranted.

## Introduction

Based on the global cancer statistics in 2020, 2.2 million people were diagnosed with and 1.8 million died of lung cancer (1). The high death rate is attributed to the advanced disease stage in approximately 65% of newly diagnosed patients (2). Early detection and diagnosis provide the best chance of a potential cure. Surgical resection (SR) and stereotactic ablative radiotherapy (SABR) are the available curative treatment options for medically operable and inoperable patients respectively with excellent treatment outcomes and prognosis when diagnosed at an early stage. Low-dose CT screening of the chest is rapidly becoming a standard of care for early detection of lung cancer (3). However, it is invasive due to radiation exposure and carries the risk of unnecessary biopsies in non-cancerous tissue (4, 5). While biopsy and/or SR provide a histopathological diagnosis, these approaches may not be feasible in patients with severe chronic obstructive pulmonary disease (COPD) or other limiting co-morbidities (6). Further, it is extremely difficult to detect cancer recurrence based on CT or PET scan post-surgery and even more challenging after SABR (7, 8). Therefore, biomarkers identified and obtained through non- invasive approaches could potentially serve as an effective therapeutic tool for early cancer detection, treatment response assessment, and cancer recurrence monitoring (4).

Metabolomics is an emerging platform with promising clinical applications in the diagnosis and management of several cancers, including lung cancer (9, 10). However, the implementation of using metabolic markers into clinical practice is limited due to variability in techniques of handling, storing, and processing the samples, as well as data analysis. Moreover, the disparity in clinical characteristics of the patients and control subjects enrolled into clinical studies poses a significant challenge in interpreting the obtained metabolomic data (11). Age, gender, race, smoking history, body mass index, physical activity and the use of over-the-counter medications can significantly influence the type and magnitude of identified metabolites (12). The current pilot study was designed to mitigate the potential mismatch between cases and controls. In this study, we present the metabolic profile of the *same patient population* before and after SR. The enrolled patients were pathologically confirmed and staged with modern imaging as early-stage non-small cell lung cancer (NSCLC). Exhaled breath condensate (EBC) and cytologically confirmed sputum samples were obtained from the *same patients* before (with cancer) and after SR (without cancer) and analyzed for any potential alterations in metabolic profile using nuclear magnetic resonance (NMR) and liquid chromatography quadrupole time-of-flight mass spectrometry (LC-QTOF-MS) techniques.

## Materials and Methods

The study protocol was approved by the University of Manitoba, Canada (H2017:247). Patients with biopsy-proven or suspected stage I/II NSCLC (based on CT/PET imaging) and presenting with surgically resectable tumors, were enrolled in the study based on exclusion/inclusion criteria described in our previously published protocol (13).

### Sputum and EBC Collection

**Figure 1:**
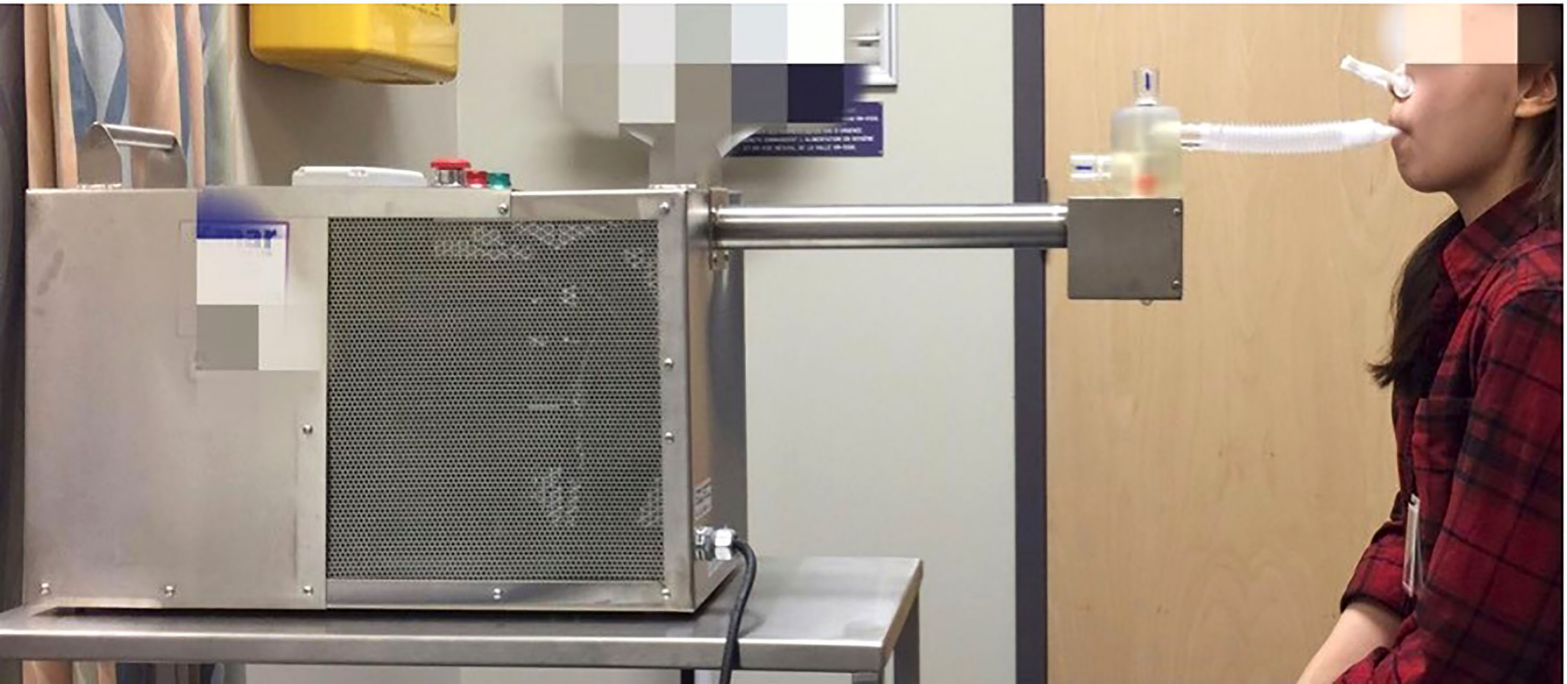
Exhaled Breath Condensate (EBC) collection. One to three ml of EBC was obtained as the patient, wearing a nose clip, exhaled into a custom-built condenser surrounding a collection tube. The patient could breathe comfortably for 20 minutes through the mouthpiece. Reprinted (Ahmed et al. 2019) (13).

The same research associate collected all the specimens. Logistic factors, including the date of the surgical procedure and the patient convenience, determined the day and time of the sample collection. One to two patients had their samples collected during the same session and on the same day. Fasting was not required as a pre-condition for biofluid sample collection. EBC and sputum samples were collected within four weeks before and within three months after SR according to a pre-established protocol (13). Sputum was collected either through a spontaneous cough or after induction by inhaled hypertonic normal saline (13). To validate that collected samples represented true sputum originating from the lower respiratory tract, only samples containing alveolar macrophages, as identified by a dedicated pathologist, were included for analysis (13–15). EBC samples were obtained by letting the patients exhale into an in-house built device as described in our previously published protocol (**Figure 1**) (13). Samples were stored at -80°C and transferred to the laboratory for NMR and LC-QTOF-MS analysis.

### NMR Analysis

Frozen EBC and sputum samples were thawed for 20–30 min. Five hundred μl of the extracted EBC was transferred into an Eppendorf tube, and 75 μl of D_2_O and 25 μl of 0.75% trimethylsilyl propanoic acid (TSP) were added. Three hundred μl of sputum was extracted and transferred into an Eppendorf tube and 300 μl of 2 M NaCl solution (pH = 7.4) and 20 μl of TSP (0.75%) were added. NMR data were obtained utilizing the Bruker software TopSpin version 3.5 pl 7, on a Bruker Ascend 600 spectrometer at 600.27 and 150.938 MHz for proton and carbon nuclei, respectively, using the specific parameters previously described (16). In summary, each sample was subject to two separate scans at a probe temperature of 298 K. A few randomly selected samples were run using the correlated spectroscopy (COSY) for additional analysis. Proton analysis with a total of 32 scans, with two dummy scans producing an acquisition time of 4.75 min, were acquired. Water was suppressed (at 2819 Hz, time domain 32.7 k) in all 1D Nuclear Overhauser Effect Spectroscopy (NOESY) runs. A total of 64 scans and 4 dummy scans were generated during the 7 min 45 s acquisition time. The 2D NMR spectra were achieved with a 90_ pulse width of 10 _s and a relaxation delay of 1.5 s. The time-domain values for 2D NMR spectra were 2048 and 400 for F1 (16 ppm) and F2 (16 ppm), respectively. An acquisition time of approximately 6h was needed to obtain 32 scans and 16 dummy scans. The Chenomx NMR suite 8.2 professional software was used to process the spectra and quantify the compounds using a database of standards. The compounds were confirmed by analysis of the 2D NMR *via* MestReNova version 12.0.0–2008.

### LC-QTOF-MS Analysis

Frozen EBC and sputum samples were thawed for 20–30 min. One hundred μl of sputum was added to 100 μl of H_2_O, vortexed at −20°C, and freeze-dried under 0.016 mbar at −53°C. Next, dried sputum was mixed with 20 μl of standard solution (15 mg/ml), 150 μl of MeOH, and 150 μl of ACN, followed by sonication for 5 min. EBC samples were transferred into a glass insert in a gas chromatography (GC) vial and subjected to LC-QTOF-MS analysis. Metabolite data was acquired on a 1260 Infinity high performance liquid chromatography (HPLC) system coupled to a 6538 ultra-high definition (UHD) Accurate Q-TOF MS and electrospray ionization source, according to the parameters established in our previously published protocol (13).

### Normalization of the data

Normalization procedure used was a ‘Percentile Shift (75%)’ which is a global normalization, where the location of all the intensities in a sample are adjusted. This normalization takes each column in an experiment independently, and computes the nth percentile of the abundance values for this sample, across all entities (where n has a range from 0-100 and n=50 is the median). It then subtracts this from the value of each entity. A baselining option (to median across samples) were also used to treat all samples equally regardless of their abundances. It subtracts the median abundance of each entity from the corresponding values in each sample.

### Statistical Analysis

Changes between classes of metabolites identified through NMR were compared using the Wilcoxon signed rank test for the comparison of two classes. The chi-square and Fisher exact tests were used to compare the frequencies of regulatory direction. Within individuals, the pre- and post-surgery levels of metabolites were compared using the Wilcoxon signed rank test for paired data.

The fold change (FC) for metabolites identified through LC-QTOF-MS was calculated by comparing average pre- and post-surgical values of each metabolite. Mass Profiler Professional (MPP; 12.6) software was used to calculate the FC (p < 0.05; ≥2-FC). Asymptotic p-values were calculated and the Benjamini–Hochberg multiple correction method was applied to identify statistically significant changes in metabolite levels. Mann–Whitney paired test and Bonferroni test were used to remove the unidentified entities. The compounds identified through LC-QTOF- MS were reconfirmed based on their mass spectra, retention time, and confidence scores against the METLIN database. Hierarchical clustering of the significant metabolites in EBC and sputum samples by heatmap was performed using XLSTAT (Addinsoft, Paris, FR Version 19.03.45028).

## Results

This study enrolled a total of 35 subjects with early-stage NSCLC. All subjects provided a pre- and post-SR EBC sample. Fifteen of the 35 subjects also provided cytologically confirmed pre- and post-SR sputum samples. Twenty of 35 subjects with cytologically unconfirmed sputum samples and/or with pre- or post-surgery sputum samples only were excluded from analysis. Minimum, median and maximum number of days for EBC collection before and after surgery were 1, 7, 31 and 16, 42 and 148 days, respectively. Minimum, median and maximum number of days for sputum collection before and after surgery were 1, 7, 31 and 23, 36, 111 days, respectively. Median time of day for sputum collection pre and post-surgery was 11.30 am and 12 noon respectively. Median time of the day for EBC collection pre and post-surgery was 11.45 am and 12.15 pm respectively. Post-SR, all but two patients had their samples taken before commencing any chemotherapy.

### Clinical Profile of Patients

Eighty three percent (25/35) of the patients were current or former smokers. Only 17% (6/35) were diabetics. None of the patients were on any oral/inhaler steroids. Eighty six percent (30/35) of all and 100% (15/15) of the patients who provided sputum samples were staged with PET scan prior to SR. Sixty percent (21/35) of the tumors were in the upper lobes of the lungs. Fifty seven percent (20/35) of the patients underwent lobectomy, 40% (14/35) wedge resection/segmentectomy, and 3% (1/35) pneumonectomy. Based on surgical pathology, all included patients had confirmed diagnosis of NSCLC. Eighty percent (28/35) of patients presented with adenocarcinoma, and 88% (30/34) had node-negative cancers. Mean tumor size was 2.7 cm and mean SUV on PET was 8.2. Programmed death ligand-1 (PDL1) and anaplastic lymphoma kinase (ALK) statuses were available in 66% of the patients (23/35). All patients (23/23) presented with ALK negative tumors and 17% (4/23) exhibited a PDL1 of >50% tumor proportion score (TPS). The clinical patient characteristics are summarized in **Table 1**. There was no statistically significant difference in characteristics of the patients who only provided EBC samples (n=20) vs. those who provided both sputum and EBC samples (n=15) (**Table 1**).

**Table 1:** Clinical characteristics of the enrolled patients.

Characteristics	Total (n = 35)	Sputum (n=15)	P
Age: mean (+/- SD) in years	64.4 (7.5)	66.7 (6.8)	0.12
Females	63% (22)	67% (10)	0.74
Males	37% (13)	33% (5)	
Smoker	34% (10)	55% (6)	0.26
Ex-smoker	52% (15)	36% (4)	
Never smoked	14% (4)	9% (1)	
Diabetes	17% (6)	13% (2)	0.68
COPD	49% (17)	53% (8)	0.74
Previous cancers	29% (10)	13% (2)	0.13
On steroids oral/inhalers	0	0	1
Squamous cell carcinoma	14% (5)	13% (2)	0.57
Adenocarcinoma	80% (28)	87% (13)	
Other	6% (2)		
Right upper lobe	29% (10)	20% (3)	0.56
Right lower lobe	20% (7)	27% (4)	
Left upper lobe	31% (11)	27% (4)	
Left lower lobe	20% (7)	27% (4)	
PET before surgery	86% (30)	100% (15)	0.31
Type of surgery			0.92
Wedge resection/segmentectomy	40% (14)	47% (7)	
Lobectomy	51% (18)	47% (7)	
Pneumonectomy	3% (1)		
Wedge and lobectomy	6% (2)	7% (1)	
Pathological stage (n = 34)			0.39
T1-T2, N0 M0	79% (27)	86% (12)	
T3-T4, N0 M0	9% (3)		
T1-T4, N1-2 M0	12% (4)	14% (2)	
Mean tumor size based on CT scan before surgical resection - mean (+/- SD) in cm	2.4 (1.6) (n = 35)	2.1 (1.1) (n=15)	0.44
Mean tumor size base on surgical pathology_ mean (+/- SD) in cm	2.7 (1.9) (n = 34)	2.3 (1.4) (n=14)	0.27
Mean maximum PET_SUV_mean (+/-SD)	8.2 (6.5) (n = 30)	7.2 (4.7) (n=15)	0.82
PDL1: < 1% (10/23)	43%	27% (3)	0.25
PDL1: 1–49% (9/23)	39%	45% (5)	
PDL1: > 50% (4/23)	17%	27% (3)	
ALK: negative (n = 23)	100%	100%	1

P-values represent the statistical significance of the comparison between patients who supplied sputum samples with those who did not (20 of 35 participants). No differences between the assessed/studied parameters were evident between these patient populations.

### Metabolic Profile of the Patients Before and After SR

One hundred and six and eighty metabolites were identified in the sputum and EBC, respectively, through LC-QTOF. After Bonferroni correction, twenty-three and nine metabolites of significance were identified in sputum and EBC, respectively. Three metabolites of statistical significance were identified in each biofluid through NMR. Metabolites (n=38) that showed a statistically significant alteration in both sputum and EBC post-SR were identified through NMR and LC-QTOF-MS (**Table 2**). Ten metabolites in EBC and 2 in sputum were either exogenous, unidentifiable, dietary supplements or related to drug metabolism and were excluded from further analysis. Metabolites included in the analysis (n=26) were re-classified based on the Human Metabolome Database (HMDB) (17) (**Table 3**).

**Table 2:** Distribution of metabolites that showed a significant alteration post-SR based on biofluid origin and the analytical platform used.

Analytical method	Biofluid sample	
	Sputum	EBC
LC-QTOF-MS	23	9
NMR	3	3
*Excluded*	*2*	*10*
Included in analysis	24	2

10 metabolites in EBC and 2 in sputum were exogenous, unidentifiable, dietary supplements or related to drug metabolism and were therefore excluded from further analysis. EBC, exhaled breath condensate; LC-QTOF-MS, liquid chromatography quadrupole time-of-flight mass spectrometry; NMR, nuclear magnetic resonance; Sputum, cytologically confirmed sputum.

**Table 3:** Twenty-six metabolites with their respective chemical classification, showing significant changes post-SR as identified in EBC and sputum samples using LC- QTOF-MS and NMR analysis.

Compound	Formula	m/z	Polarity	FC	p Value	Reg	Class	Biofluid	Platform
Lipids									
2-Arachidonylglycerol	C23H38O4	757.5556	Pos	11	0.0032	Up	Endocannabinoids	Sputum	MS
2-Hydroxyhex-adecanoylcarnitine	C23H45NO5	454.2928	Pos	7	0.039	Up	Fatty acid ester	Sputum	MS
Deoxycholic acid glycine conjugate	C26H43NO5	899.6339	Pos	6	0.0351	Down	Bile acid	Sputum	MS
Docosatrienoic acid	C22H38O2	707.5414	Pos	7	0.0043	Up	Fatty acids	Sputum	MS
Eicosenoic acid				20	0.0001	Up	Fatty acids	Sputum	MS
L-Acetylcarnitine	C9H17NO4	407.2284	Pos	9	0.0063	Up	Acyl carnitines	Sputum	MS
LysoPC (P-16:0)	C24H50NO7	991.6712	Pos	7	0.0066	Up	Glycerophopholipids	Sputum	MS
Methylmalonyl-carnitine	C11H19NO6	279.1589	Pos	10	0.0008	Up	Acyl carnitines	Sputum	MS
PS (18:1/18:2)	C42H76NO10	803.5431	Pos	9	0.0046	Up	Glycerophopholipids	Sputum	MS
Retinyl beta-glucuronide	C26H38O7	480.3085	Pos	10	0.0012	Up	Lipid	Sputum	MS
TG (16:0/16:0/18:1)	C53H100O6	871.7360	Pos	11	0.0001	Down	Triacylglyceride	Sputum	MS
TG (16:0/18:1/18:1)	C55H102O6	859.7714	Pos	12	0.0001	Down	Triacylglyceride	Sputum	MS
TG (16:1/18:2/20:0)	C57H104O6	885.7835	Pos	7	0.0189	Down	Triacylglyceride	Sputum	MS
TG (18:2/18:0/18:3)	C57H100O6	881.7565	Pos	8	0.0002	Down	Triacylglyceride	Sputum	MS
Carboxylic Acid									
Acetate	C2H4O	–	–	*0.4	0.015	Down	Carboxylic acid	Sputum	NMR
Dodecanedioylcarnitine	C19H35NO6	764.5251	Pos	14	0.0005	Up	Carboxylic acids and derivatives	Sputum	MS
Propionate	C3H5O2	–	–	*0.02	0.020	Down	Carboxylic acid	Sputum	NMR
			Carbohydrate						
Glucose	C6H12O6	–	–	*0.01	0.037	Up	Carbohydrate	Sputum	NMR
Purine									
6-Dimethylaminopurine	C7H9N5	365.1356	Pos	9	<0.0001	Down	Purines	Sputum	MS
Adenosine monophosphate	C10H14N5O7P	348.0685	Pos	13	0.0037	Up	Purine nucleotide	Sputum	MS
Others									
4-Hydroxy cyclohexylcarboxylic acid	C7H12O3	162.1121	Pos	10	0.0031	Up	Cyclohexanol	Sputum	MS
Acetoin	C4H8O2	–	–	*0.001	0.043	Up	Acyloin	EBC	NMR
D-myo-Inositol-1,3,4,5-tetraphosphate	C6H16O18P4	498.9151	Neg	7	0.023	Up	Onositol phosphate	EBC	MS
L-Carnitine	C7H15NO3	345.1995	Pos	11	0.0021	Up	Carnitine	Sputum	MS
N1,N12-Diacetylspermine	C14H30N4O2	287.2439	Pos	8	0.011	Up	Carboximidic acid	Sputum	MS
Nicotine imine	C10H13N2	345.2048	Pos	12	0.0021	Up	Alkaloid	Sputum	MS

EBC, exhaled breath condensate; FC, log fold change; m/z, mass to charge ratio; Reg, up- or downregulated; * Median fold change in concentration.

### Pattern of Dysregulation Post SR

**Figure 2:**
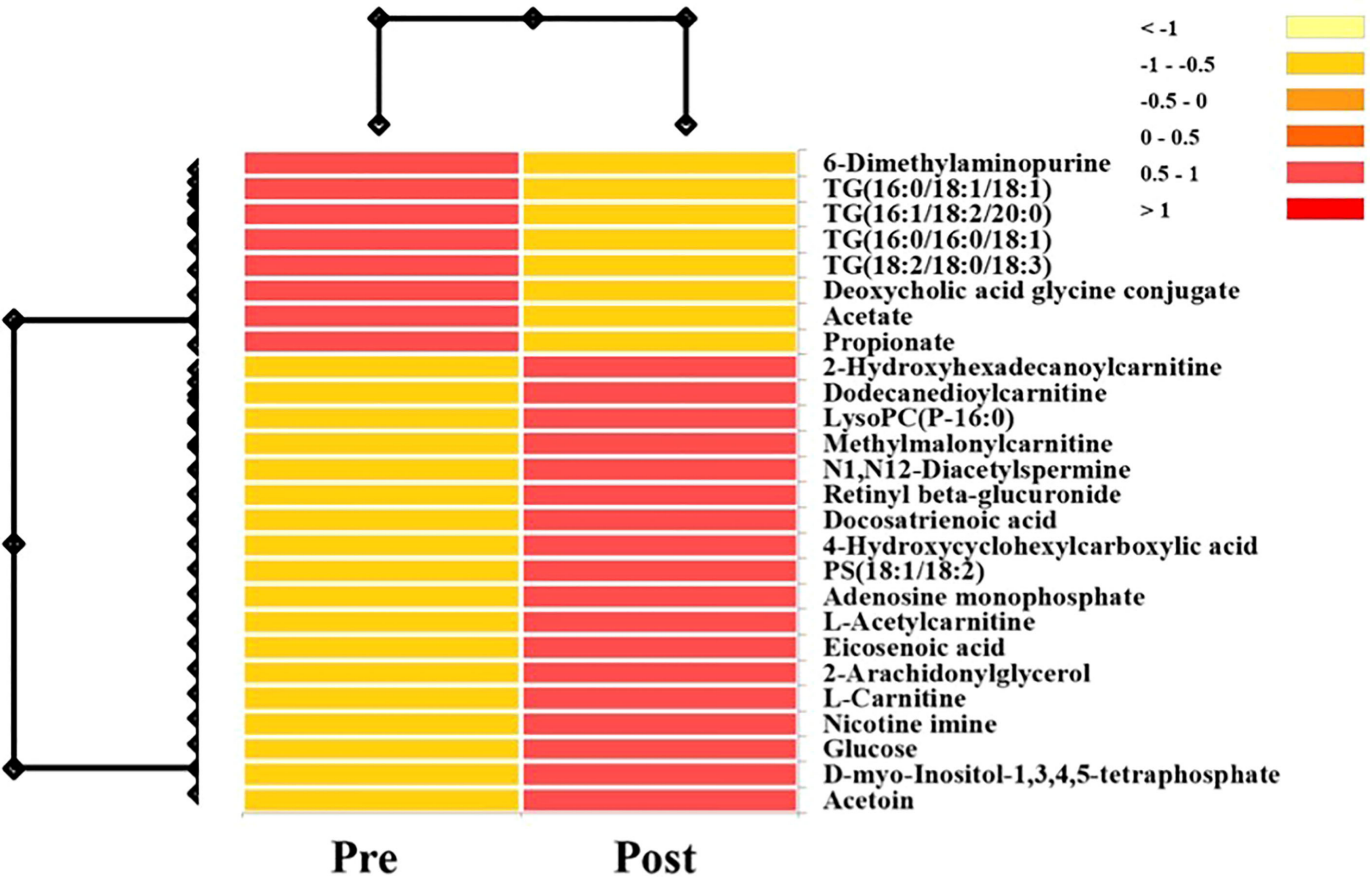
Hierarchical heatmap summarizing the 26 significant metabolites (Paired T-Test; P<0.05) between pre and post-surgical resection in sputum and EBC and using LC-MS-QTOF and NMR methods. Concentration of the individual metabolites range between (log 2) <1 to >1.

**Figure 3:**
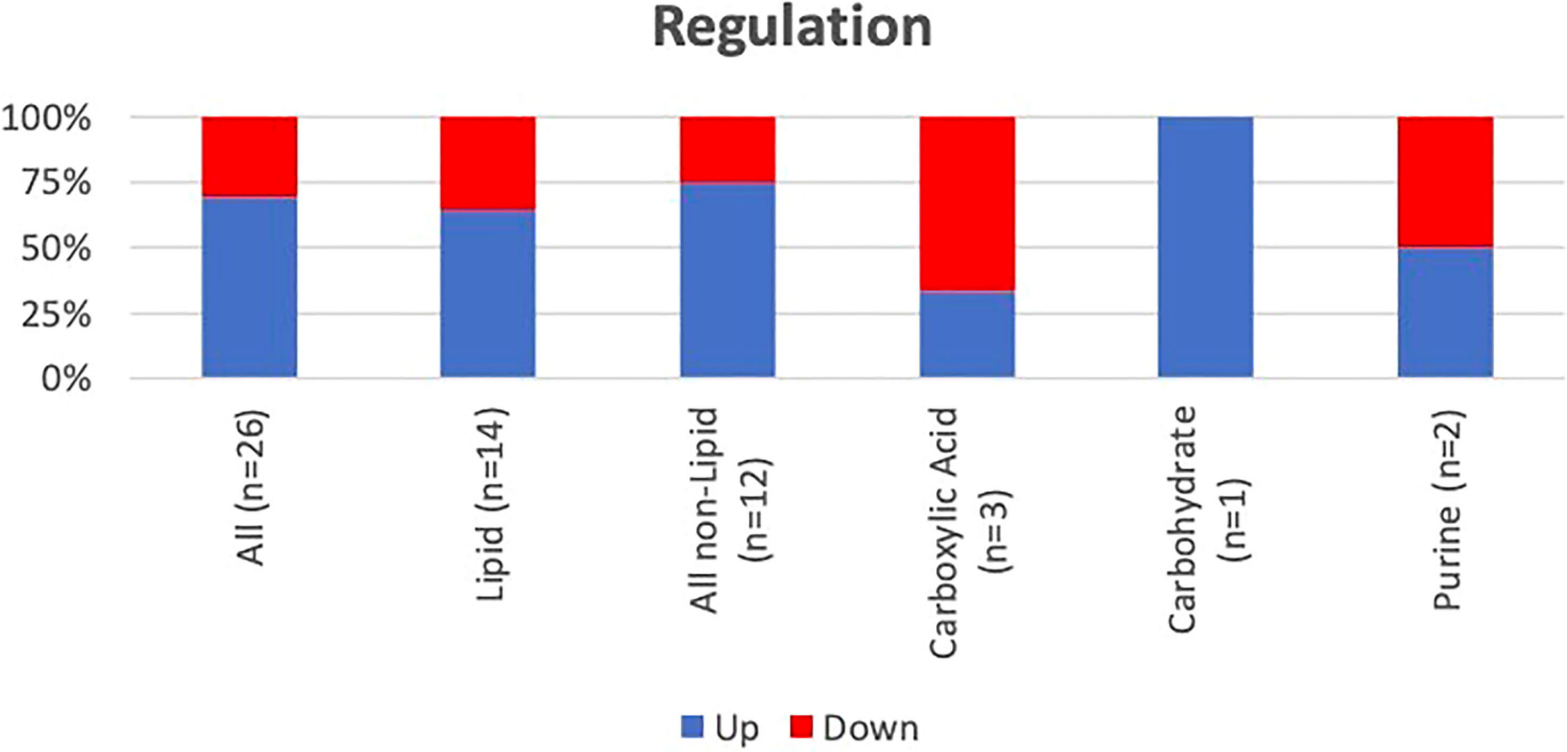
Frequency and pattern of alteration post-SR of the identified metabolites. Lipids and other compounds represent 54% and 46% of the identified metabolites. There was no difference in pattern of dysregulation (frequency of up vs down) of the lipids (p=0.42) or the other compounds (p=0.15).

A hierarchical heatmap summarizing the 26 significant metabolites (paired t-test; p<0.05) between pre and post-surgical resection in sputum and EBC and using LC-MS-QTOF and NMR methods is presented in **Figure 2**. Concentration of the individual metabolites ranged between (Log 2) < 1 to > 1. The metabolites were classified as lipids, carboxylic acids, carbohydrates, purines, inositol phosphates, acyloins, carboximidic acid, cyclohexanols, alkaloids and carnitine. Fourteen of 26 metabolites (54%) were lipids; the remaining compounds belonged to nine different classes of metabolites as summarized in **Figure 3**. Of the 26 metabolites,18 were upregulated (69%) and 8 (31%) were downregulated with no statistically significant difference in frequency of dysregulation (up-down) post SR. (p= 0.08). Similarly, among the lipids, 9 (64%) were upregulated and the remaining 5 (36%) were downregulated with no statistically significant difference in frequency of dysregulation (up/down) post SR (p= 0.42).

### Magnitude of Dysregulation Post SR

**Figure 4:**
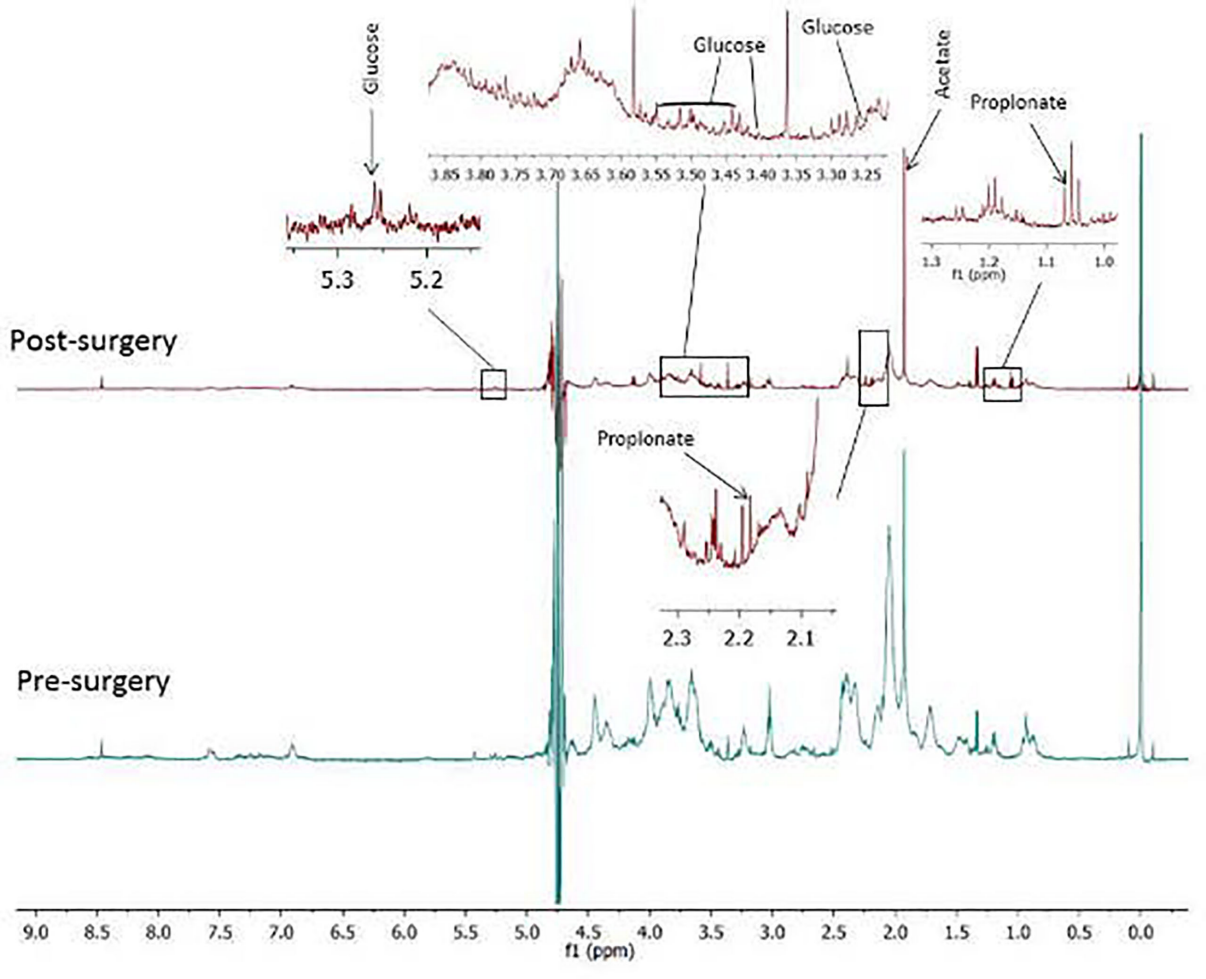
Proton NMR spectra of sputum containing glucose, acetate and propionate before and after surgical resection (SR) of early stage non-small cell lung cancer. While glucose in sputum increased after SR, acetate and propionate decreased post SR. .

Median, maximum, and minimum FCs for all the up- and downregulated metabolites post-SR are presented in **Table 4**. The median FCs for the up- and downregulated metabolites identified through LC-QTOF were 10 and 8, respectively with no statistically significant difference in the magnitude of dysregulation (up-down) post SR (p=0.30). The respective median fold change in concentration for the up- and downregulated metabolites identified through NMR were 0.04 and 0.27 with no statistically significant difference in the magnitude of dysregulation (up-down) post SR (p=0.72). Proton NMR spectra of the sputum displaying metabolites of significance are presented in **Figure 4**.

**Table 4:** Magnitude of dysregulation of all the identified metabolites analyzed using LC-QTOF and NMR.

Class of Metabolites	Upregulated				Downregulated				p Value
	n	Med FC	Min FC	Max FC	n	Med FC	Min FC	Max FC	
All LC-QTOF-identified metabolites	16	10	7	20	6	8	6	12	0.30
Lipid LC-QTOF-identified metabolites	9	9	7	20	5	8	6	12	0.70
All NMR-identified metabolites	2	0.040	0.002	0.078	2	0.27	0.068	0.48	0.72

There was no statistically significant difference in log-fold change (LC-QTOF) or median fold change in concentration (NMR) between up- and downregulated metabolites post-SR. FC, fold change; Max, maximum; Med, median; Min, minimum.

### Metabolites of Potential Clinical Significance

Glucose, adenosine monophosphate and N1, N12-diacetylspermine were identified in sputum and have previously been reported to be of clinical significance (15, 18–23). These molecules were upregulated post-SR. Glucose levels (assessed by NMR) were either undetectable or very low before surgery and increased significantly post-SR with a median fold change of 0.01 (p = 0.037). Adenosine monophosphate and N1, N12-diacetylspermine (determined by LC-QTOF) were upregulated by 13- (p=0.0037) and 8-fold (p=0.011), respectively, post-SR.

## Discussion

NMR and LC-QTOF-MS are common technical analytical platforms used to identify metabolites in body fluids. While NMR is nondestructive and can rapidly detect multiple metabolites with minimal sample preparation, LC-QTOF-MS has the advantage of detecting metabolites in very low concentrations (24, 25). NMR is especially useful to detect molecules related to glycolysis and the tricarboxylic acid (TCA) cycle. However, there are limitations to the NMR approach. For example, it can only detect 20–50 metabolites, whereas MS (especially LC-MS) can detect hundreds or even a thousand metabolites from a single biofluid sample (26–28). Several body fluids, including blood serum and plasma, urine, sputum, and EBC, have been studied for metabolic alterations caused by cancer using these technical platforms (10, 29). We have previously demonstrated a relative absence of glucose in cytologically confirmed sputum of patients with locally advanced NSCLC using NMR, thereby identifying glucose as a potential biomarker of lung cancer (15). Recently, we reported significant alterations in the major metabolic pathways identified in blood and urine of early-stage NSCLC patients (after SR of their tumors) using NMR and LC-QTOF-MS (16).

The current study evaluated the metabolic profile of medically operable early-stage lung cancer patients undergoing curative SR. The specific design of this pilot study was unique as *controls* and *cases* belong to the same patient population (i.e., each patient served as his/her own control); in other words, SR provided two distinct metabolomic states of the same patient population *with* or *without* cancer. A similar study design was used to identify urinary metabolites to detect recurrent non-muscle-invasive bladder cancer (30). We prospectively collected metabolic data from EBC and sputum samples obtained from the same patient population before and after SR. Both sputum and EBC samples were obtained, before SR (median time,7days) and after SR (median time 36-42days). All patients who provided EBC or EBC and sputum exhibited similar clinical, radiological, pathological and genomic characteristics, and underwent similar surgical procedures. It was challenging to obtain cytologically confirmed sputum from the study participants; samples obtained from 29% (10/35) of the patients who provided both pre- and post-surgery samples could not be cytologically confirmed as sputum and were therefore excluded from further analysis. However, the number of identified metabolites of significance (i.e., those of which levels were altered post-SR) was higher in sputum (n=24) than in EBC (n=2; **Table 3**). Although this pilot study features a relatively small sample size, the results likely represent the biological and metabolic state of the lower respiratory tract of early-stage NSCLC patients before and after SR of the cancerous tumors.

Metabolic alterations were most pronounced with regards to lipid metabolism as compared to other classes of metabolites, which is consistent with previous publications (16, 31). However, there was no specific pattern of dysregulation; the magnitude of up- and downregulated lipid metabolites was similar (**Figure 3** and **Table 4**). Among the identified lipids, the endocannabinoid 2-arachidonylglycerol was upregulated by 11-fold post-SR. The endocannabinoid system is comprised of cannabinoid receptors, cannabinoid ligands and their signaling network, and has been associated with different tumor states. As such, it represents a potential molecular platform for the development of pharmacological anti-cancer agents (32, 33). In line with our findings, increased levels of 2-arachidonylglycerol have been observed in human endometrial carcinoma (34).

Adenosine monophosphate belongs to the purine ribonucleoside monophosphate class (17) and was upregulated by 13-fold post-SR. This significant biochemical change is possibly related to the surgical removal of the tumor. The metabolism in cancerous cells is anaerobic; thus, cells consume more glucose/oxygen, produce less ATP, and, as a result, less AMP. After tumor removal, healthy lung cells catabolize glucose *via* aerobic pathways, producing more ATP and, consequently, more AMP. AMP-activated protein kinase (AMPK) plays a central role in regulation of cellular metabolism and energy homeostasis, and other cellular processes including apoptosis and inflammation (35, 36). Furthermore, a correlation between adenosine monophosphate kinase (AMPK) activity and lung tumor growth has been proposed. AMPK activation leads to inhibition of processes involved in cell proliferation, including the synthesis of fatty acids, phospholipids, proteins, and ribosomal RNA. There is evidence to suggest that NSCLC cells have an acquired liver kinase B mutation, which suppresses AMPK activation and promotes the proliferation of cancer cells (20).

N1, N12-diacetylspermine (DASr) belongs to a class of carboximidic acids, is a component of polyamine metabolism in humans, and was found to be increased by 8 log-fold in sputum samples. Actively proliferating cells excrete more polyamines due to activation of intracellular polyamine metabolism, which is increased in advanced stage cancers (21). Results from a large study in Japan, consisting of 260 NSCLC patients and 239 controls (participants with benign diseases and/or healthy volunteers), showed that urinary DASr was significantly higher in NSCLC patients (0.810 vs 0.534, P=0.0111), and that the alteration was more pronounced in squamous cells than in adenocarcinoma (23). The same group of investigators have also demonstrated a significant correlation between urinary DAcSr levels and pathological tumor invasiveness in early-stage NSCLC (22). Based on our current results and these previous observations by others, we strongly believe this molecule could represent a promising biomarker of lung cancer; further investigations in larger clinical cohorts are warranted for validation

We demonstrate that glucose was upregulated in sputum post-SR. This finding is consistent with published data and confirms our previous observations in locally advanced NSCLC (15, 19). In a small group of patients, Bezabeh *et al.* demonstrated an absence of glucose in sputum of locally advanced NSLC patients compared to samples from participants without cancer (18).

Acetate was found to be downregulated in sputum after SR. Recently published data suggest an important role for this molecule as a respiratory substrate and a source of nutrition to cancer cells supporting the growth of several human cancers. Acetyl-CoA synthetase promotes the conversion of acetate to acetyl-CoA plays and has been found to play a significant role in the growth of hepatocellular carcinoma, glioblastoma, breast cancer and prostate cancer. A mechanism that will reduce or prevent the re-uptake of acetate by cancer cells may provide a therapeutic opportunity in the treatment of cancers with altered acetate metabolism (15, 37, 38).

Acetoin was upregulated in EBC after SR. Altered levels of acetoin, as in the samples of our patients, have also been observed in EBC of pancreatic cancer patients (39).

D-myo-inositol-1,3,4,5-tetraphosphate detected in EBC was upregulated by 7 log-folds after SR. This molecule has been found in human blood and is a human exposome (40). Primarily an inositol phosphate and a carbohydrate molecule, it is importantly involved in energy homeostasis, but also serves as an anti-oxidant and anti-inflammatory compound. It modulates mRNA transcription, chromatin remodeling, and cytoskeleton configuration, with a potential role in inhibition of the malignant process (41, 42). Myo-inositol metabolism is also regulated by P53, which is a tumor suppressor gene (43). Decreased inositol levels in lung cancer and cerebral astrocytoma have been observed. Accordingly, exogenous inositol (myo-inositol or IP6) can inhibit cancer cell growth (44–46). It is unknown if the patients in this study were exposed to this compound or its derivatives.

L-carnitine, a non-essential amino acid and carnitine, was found to be upregulated by 11-fold post-SR. L-carnitine is also a mitochondrial metabolite involved in fatty acid β-oxidation in mitochondria, and represents a potential biomarker and target for drug discovery in lung cancer (47).

Propionate, a carboxylic acid and another mitochondrial metabolite, was downregulated post-SR. It has been suggested that propionate may have an immune anti-cancer activity through expression of natural killer group 2D (NKG2D) ligand in cancer cells (48). We have previously observed lower concentrations of propionate in the sputum of advanced lung cancer patients compared to subjects with benign diseases (15).

## Strengths and Limitations of the Study

The authors acknowledge that there are several limitations of this pilot study. The relatively small sample size influencing the statistical power of the observed metabolite data is a primary concern. Further, NMR and LC-QTOF used in this study are highly complex analytical approaches, typically not available for routine laboratory investigations in clinical practice.

However, the unique design of our study in which sputum and EBC samples were obtained from the same patient population before and after complete SR of the cancer, provides an opportunity to directly compare the pre- and post-SR metabolic states uninfluenced by the heterogeneity of patients or tumor characteristics, which have been major challenges in the interpretation and validation of previously published data (11). Moreover, in contrast to radiotherapy or systemic therapy where treatment may or may not eliminate the disease completely, in the current study, all patients underwent SR with either none or minimal microscopic disease demonstrated in their post-surgical pathology. Additional cytological confirmation of the sputum further validated the observed metabolic data. The patients enrolled in this study represent early-stage lung cancer with small tumors and minimal tumor burden. Therefore, findings from this study may provide a basis for further exploration of non-invasive tools for early-stage metabolomic lung cancer screening

## Conclusions

The current pilot study presents the changes in the metabolic profile of patients with early-stage NSCLC pre- and post-SR. The identified metabolites altered up to 8-10-fold post SR. However, we did not observe any specific trend of dysregulation of metabolites (up vs. down) post-SR. In addition, we demonstrated that non-invasive biofluid (EBC and sputum) sample collection from patients was feasible. EBC was convenient to obtain but only provided limited metabolic yield when analyzed with NMR and LC-QTOF. On the other hand, while it was necessary to cytologically confirm sputum, the observed metabolic profile in sputum was much more robust and of potential clinical significance. Among all the identified classes of metabolites, alterations of lipid metabolism were most pronounced. We have observed several metabolites of clinical interest in this study. However, based on the previously published data, the most significant findings of this study are: 1) the absence/decreased levels of glucose in sputum before surgery and its subsequent rise post-SR, and 2) the identification of dysregulation in adenosine monophosphate and N1, N12-diacetylspermine levels post-SR. We believe these molecules may be indices of a pre-or an early malignant process and could serve as a promising non-invasive tool for early diagnosis of NSCLC. In addition, the evaluation of individual metabolic signatures could facilitate the identification of targets to monitor response to treatment and personalized pharmacological intervention. Clinical trials with a larger sample size are required to validate these observations.

## Data Availability Statement

The original contributions presented in the study are included in the article/supplementary material. Further inquiries can be directed to the corresponding authors.

## Ethics Statement

The study was conducted according to the guidelines of the Declaration of Helsinki. The study was reviewed and approved by the Institutional Review Board of the University of Manitoba, protocol code H2017:247, approved 31 July 2017. The patients/participants provided their written informed consent to participate in this study.

## Author Contributions

Author Contributions: Conceptualization, NA, MA, and BK; methodology, NA, BK, and MA; software, MA; validation, NA and MA; formal analysis, MA, NA, and ZN; investigation, NA, MA, LW, and NM; resources, RM, MA, BK, LT, GB, SS, AM, SS-I, and GQ; data curation, NA and ZN; original draft preparation, NA; manuscript review and editing, NA, MA, BK, NM, and LW; supervision, NA; project administration, NA, MA, and LW; funding acquisition, NA, BK, RM, and MA. All authors contributed to the article and approved the submitted version.

## Funding

We wish to acknowledge and thank CancerCare Manitoba Foundation for their generous funding to this project (grant number 761075015).

## Conflict of Interest

The authors declare that the research was conducted in the absence of any commercial or financial relationships that could be construed as a potential conflict of interest.

## Publisher’s Note

All claims expressed in this article are solely those of the authors and do not necessarily represent those of their affiliated organizations, or those of the publisher, the editors and the reviewers. Any product that may be evaluated in this article, or claim that may be made by its manufacturer, is not guaranteed or endorsed by the publisher.
